# Utility of a custom designed next generation DNA sequencing gene panel to molecularly classify endometrial cancers according to The Cancer Genome Atlas subgroups

**DOI:** 10.1186/s12920-020-00824-8

**Published:** 2020-11-30

**Authors:** Eirwen M. Miller, Nicole E. Patterson, Gregory M. Gressel, Rouzan G. Karabakhtsian, Michal Bejerano-Sagie, Nivedita Ravi, Alexander Maslov, Wilber Quispe-Tintaya, Tao Wang, Juan Lin, Harriet O. Smith, Gary L. Goldberg, Dennis Y. S. Kuo, Cristina Montagna

**Affiliations:** 1 Division of Gynecologic Oncology, Department of Obstetrics and Gynecology and Women’s Health, Montefiore Medical Center, Bronx, NY 10461 USA; 2 Department of Genetics, Albert Einstein College of Medicine, Price Center/Block Research Pavilion, Room 401, 1301 Morris Park Avenue, Bronx, NY 10461 USA; 3 Department of Pathology, Montefiore Medical Center, Bronx, NY 10461 USA; 4 Department of Epidemiology and Population Health, Albert Einstein College of Medicine, Bronx, NY 10461 USA; 5 Present Address: Department of Obstetrics and Gynecology, Northwell Health, LIJ Medical Center, New Hyde Park, NY 11040 USA

**Keywords:** Endometrial carcinoma, Uterine serous carcinoma, Endometrioid uterine carcinoma, Next generation sequencing, Genomic profiling, Molecular groups, Targeted sequencing

## Abstract

**Background:**

The Cancer Genome Atlas identified four molecular subgroups of endometrial cancer with survival differences based on whole genome, transcriptomic, and proteomic characterization. Clinically accessible algorithms that reproduce this data are needed. Our aim was to determine if targeted sequencing alone allowed for molecular classification of endometrial cancer.

**Methods:**

Using a custom-designed 156 gene panel, we analyzed 47 endometrial cancers and matching non-tumor tissue. Variants were annotated for pathogenicity and medical records were reviewed for the clinicopathologic variables. Using molecular characteristics, tumors were classified into four subgroups. Group 1 included patients with > 570 unfiltered somatic variants, > 9 cytosine to adenine nucleotide substitutions per sample, and < 1 cytosine to guanine nucleotide substitution per sample. Group 2 included patients with any somatic mutation in *MSH2*, *MSH6*, *MLH1*, *PMS2*. Group 3 included patients with *TP53* mutations without mutation in mismatch repair genes. Remaining patients were classified as group 4. Analyses were performed using SAS 9.4 (SAS Institute Inc., Cary, North Carolina, USA).

**Results:**

Endometrioid endometrial cancers had more candidate variants of potential pathogenic interest (median 6 IQR 4.13 vs. 2 IQR 2.3; *p* < 0.01) than uterine serous cancers. *PTEN* (82% vs. 15%, *p* < 0.01) and *PIK3CA* (74% vs. 23%, *p* < 0.01) mutations were more frequent in endometrioid than serous carcinomas. *TP53* (18% vs. 77%, *p* < 0.01) mutations were more frequent in serous carcinomas. Visual inspection of the number of unfiltered somatic variants per sample identified six grade 3 endometrioid samples with high tumor mutational burden, all of which demonstrated *POLE* mutations, most commonly P286R and V411L. Of the grade 3 endometrioid carcinomas, those with *POLE* mutations were less likely to have risk factors necessitating adjuvant treatment than those with low tumor mutational burden. Targeted sequencing was unable to assign samples to microsatellite unstable, copy number low, and copy number high subgroups.

**Conclusions:**

Targeted sequencing can predict the presence of *POLE* mutations based on the tumor mutational burden. However, targeted sequencing alone is inadequate to classify endometrial cancers into molecular subgroups identified by The Cancer Genome Atlas.

## Background

Endometrial cancer is the fourth most common malignancy in women and the most common gynecologic cancer in the United States, with an estimated 61,880 new diagnoses and 12,160 cancer-related deaths in 2019 [1]. Traditionally, endometrial cancer is divided into two histologic subtypes: endometrioid (type 1) endometrial cancers (EAC) which are thought to be driven by estrogen exposure unmitigated by progestins, and serous (USC) and clear cell (type 2) endometrial cancers. Type 1 endometrial cancers often present with low grade and early stage tumors and are usually associated with a favorable prognosis. Though type 1 tumors account for greater than 80% of diagnosed endometrial cancers, they represent only about 50% of the cancer-related deaths [2]. Comparatively, type 2 tumors are not considered to be hormonally driven, present as high grade, late stage lesions with a far less favorable prognosis. While type 2 endometrial cancers represent less than 10% of all uterine cancers, they are responsible for approximately 20–39% of cancer-related deaths [2, 3]. These histologic categories and their associated phenotypic differences guide current adjuvant treatment protocols. However, clinical responses to standard platinum-based chemotherapy regimens vary significantly between these patients [4]. Therefore, tools that aide in additional stratification and pre-treatment identification of markers of poor responders are needed.

A growing body of evidence suggests that future directions of cancer care lie in targeted therapies [5–8]. For example, in a study of 86 patients with mismatch repair (MMR) deficient cancers, including endometrial cancer, *PD-1* blockade with pembrolizumab resulted in 77% disease control rate [9]. Similarly, in a study of platinum sensitive recurrent ovarian cancer, maintenance treatment with niraparib was associated with 16-month progression free survival benefit, compared with placebo, in patients with a germline *BRCA* mutation [10]. In these studies, genomic signatures predicted tumor phenotype and treatment responses above and beyond the anatomic site of the disease or the histologic classification. These studies, and others, have changed the standard of care in oncology across disease sites. The goal of targeted mutational testing is to identify pathogenic somatic genetic changes [e.g. single nucleotide variants (SNVs), insertions and deletions (INDELs), copy number variations, methylation changes] driving tumorigenesis which may influence the efficacy of therapeutic interventions [11]. In efforts to study tumors at the genomic level, next generation sequencing (NGS) technologies have been developed for whole genome (WGS), whole exome (WES), transcriptome, and targeted gene sequencing. The Cancer Genome Atlas (TCGA) utilized WGS and WES techniques to compile genetic data on many tumor types [12], including endometrial cancer [13] and provides an exceptional validated resource to begin investigating the mutational spectrum of tumors of specific sites and histology.

In 2013, TCGA published genomic and proteomic data from 373 endometrial carcinomas [13]. The collected data allowed for reclassification of endometrial cancers into four categories: (1) *POLE* ultra-mutated, (2) microsatellite instability (MSI) hypermutated, (3) microsatellite stable copy number low, and 4) copy number high. Most EACs (comprising clusters 1, 2, and 3) were characterized by few copy number alterations and few *TP53* mutations but frequent mutations in *PTEN*, *CTNNB1*, *ARID1A*, *ARID5B*, and *KRAS*. Comparatively, cluster 4, comprising 94% of USCs and 12% of EACs, demonstrated extensive copy number alterations of oncogenes *MYC*, *ERBB2*, and *CCNE1*, and frequent *TP53* mutations. Correlations between cluster type and progression free survival were identified: the ‘serous-like’ cluster 4 was associated with poor prognosis indicating that 12% of EACs may be associated with a more aggressive clinical phenotype than predicted by tumor histology alone [13].

This data from TCGA suggests that the stratification of patients based on tumor genomic signatures could augment or replace the more simplistic dualistic model and assist in individualizing therapy. Knowledge of a tumor’s genetic profile may help to individualize chemotherapy and other therapeutic regimens. A randomized phase III clinical trial comparing adjuvant radiation and chemotherapy with radiation alone in patients with high risk endometrial cancer used *p53* and MMR immunohistochemistry and *POLE* sequencing to define 4 molecular subgroups (similar to TCGA) and demonstrated differences in treatment response across groups. Patients with *p53* abnormal tumors that received combined adjuvant chemotherapy and radiation had a 50% reduction in 5-year recurrence risk compared with those that received radiation alone. However, there was no survival advantage with the addition of chemotherapy to radiation in patients with *POLE* mutations or MMR deficiency [14]. Additionally, current phase III clinical trials are investigating the utility of adding pembrolizumab or dostarlimab (*PD-1* blockade) to standard of care chemotherapy in patients with MMR deficient advanced endometrial cancer (NCT03914612 and NCT03981796). Similarly, phase 2 data demonstrate a 9-month progression free survival benefit with addition of trastuzumab (Anti-*HER2/neu* monoclonal antibody) to standard of care chemotherapy in patients with endometrial cancers that overexpress *HER2/neu* [15].

Although WGS and WES, as utilized by TCGA, provide power to dissect the complexity of the whole cancer genome, the application of these approaches in the clinical setting is limited by sequencing cost and time. In addition, WES generally provides only ~ 30X coverage, which limits understanding of tumor clonality. We designed a cancer focused targeted gene panel for the identification of single nucleotide variants (SNVs) and insertions and deletions (INDELs) in 156 genes, frequently mutated in gynecologic, breast, colon, gastrointestinal, and lung cancers. By providing ~ 500× coverage, our gene panel detects mutations with higher sensitivity than WGS at more affordable cost [16], making it a viable option for application in the clinical setting. Our primary objective was to test the hypothesis that this high-depth targeted sequencing panel alone, without adjuvant testing (WGS, copy number variation, immunohistochemistry, microsatellite instability assessment), could identify tumor specific genetic signatures that allow for molecular classification of endometrial cancer according to the 4 TCGA subgroups.

## Methods

Approval to perform this study was obtained from the Montefiore Medical Center/Albert Einstein College of Medicine Institutional Review Board (IRB #2019-10496). We retrieved 56 endometrial cancer tumor (T) specimens and matching non-tumor (NT) tissue from the Montefiore Medical Center Gynecologic Oncology Biorepository. A single representative hematoxylin and eosin (H&E) stained section of each sample (T and matched NT) was reviewed by an expert gynecologic pathologist to confirm appropriate classification. When T and NT were present in a single specimen, the tumor was outlined on the H&E stained slide. Ten serial 5 µM sections were prepared on unstained slides for each frozen specimen. Areas of tumor, as identified and outlined in the H&E stained slide, were subsequently grossly micro-dissected from the frozen unstained sections using a scalpel for DNA extraction using the QIAamp DNA Mini kit (Qiagen, Hilden, Germany) according to the manufacturer’s protocol. The DNA concentration of each sample was determined using the Qubit dsDNA HS Assay kit (Thermo Fisher Scientific, Waltham, MA), according to the manufacturer’s protocol.

### Targeted next generation sequencing (NGS)

All samples were sequenced using the Einstein Custom Cancer Panel (ECCP), a custom-designed targeted next generation sequencing (NGS) panel of 156 oncogenes and tumor suppressor genes, designed to investigate somatic and germline genomic alterations common to a variety of solid tumors, including breast and gynecologic malignancies [16]. The ECCP has been extensively studied and validated with both breast and endometrial cancer samples [16]. Samples were prepared for targeted sequencing as previously reported [16]. Target genes were amplified in two separate 10 µL reactions (one for each of two primer pools) for each sample by combining 10 ng of input DNA, the Ion AmpliSeq Einstein Custom Cancer Panel amplification primers, and reagents from the Ion AmpliSeq Library Preparation kit 2.0 (Thermo Fisher Scientific, Waltham, MA) according to the manufacturer’s protocol. Following amplification, the DNA libraries were combined with FuPa reagent provided in the Ion AmpliSeq Library Preparation kit 2.0 (Thermo Fisher Scientific, Waltham, MA). To allow multiplex sequencing of the samples, each sample was then ligated to a uniquely barcoded IT Xpress adapter. The libraries were purified using 1.5X Agencourt AMPure XP kit (Beckman Coulter Inc, Brea, CA), eluted in 50 µL low TE and diluted 1:100 before mixing with either the Applied Biosystems’ Ion Library Quantitation kit or KAPA Biosystems’ Ion AmpliSeq Library Quantitation kit. All libraries were quantified using the Applied Biosystems StepOne Plus real-time qPCR system and pooled together after being diluted to 100 pM in preparation for sequencing. Libraries were only sequenced simultaneously if they had been quantified using the same quantification kit (Applied or KAPA). The Ion OneTouch 2 system (Thermo Fisher Scientific, Waltham, MA) was used to amplify the library fragments onto Ion Sphere Particles (ISPs) provided with the Ion PI Template OT2 200 kit v2 (Thermo Fisher Scientific, Waltham, MA). The Ion Sphere Quality Control kit with the Qubit 2.0 fluorometer (Thermo Fisher Scientific, Waltham, MA) was used to assess the template efficiency of ISPs, ensuring that the percent of templated ISPs was between 10%-30%. Sequencing was performed on the Ion Proton platform (Thermo Fisher Scientific, Waltham, MA), using PI sequencing chip (Thermo Fisher Scientific, Waltham, MA) and Ion PI Sequencing 200 kit v3 (Thermo Fisher Scientific, Waltham, MA), according to the manufacturer’s protocol.

Twelve hypermutated specimens were identified and further analyzed for mutations in *POLE*. As *POLE* is not included in the Einstein Custom Cancer Panel, Ion AmpliSeq Designer v6.0, we custom-designed an assay for the *POLE* gene using Human Genome version 19 (hg19). The assay covers 11.19 kb in 108 amplicons, split into two primer pools. The panel design, which is also suitable for use with Formalin-Fixed Paraffin Embedded (FFPE) derived DNA and both somatic and germline applications*,* has 92.74% coverage of the exonic regions of the *POLE* gene (Additional file 1: Table S1). Libraries were prepared from 10 ng input DNA per primer pool using the Ion AmpliSeq Library Preparation kit 2.0 according to the manufacturer’s instructions. For amplification, 19 PCR cycles, and an anneal/extension time of 4 min were used, and for ligation, samples were barcoded with NEB NextFlex adapters. Quality control and sequencing were performed as outlined above, multiplexing a total of 64 libraries.

### NGS data analysis: identification of all somatic variants

Following sequencing, the reads from each sample were aligned to the NCBI human reference genome (hg19-Genome Reference Consortium GRCh37). Aligned reads were analyzed using Ion Torrent Suite’s Torrent Variant Caller v4.4 (TVC) for the Einstein Custom Cancer Panel and v5.6 (TVC) for *POLE*. According to the company’s recommendation for custom panels, low stringency parameters were applied by the Variant Caller package. The variant files were subsequently imported into Ion Reporter Software version 4.4 (Thermo Fisher Scientific, Waltham, MA) for variant calling and annotation. Using a paired (two sample) workflow in the Ion Reporter Software, with company standard filtering parameters, variants from each T and matching NT specimen were compared to identify somatic variants for each sample (herein defined as unfiltered somatic variants). Identified variants were classified either single nucleotide variants (herein defined as unfiltered somatic SNVs) or INDELs (herein defined as unfiltered somatic INDELs) and single nucleotide variants were grouped by nucleotide shift [cytosine (C) > adenine (A), cytosine (C) > guanine (G)].

Top or bottom 25% quantile of number of unfiltered somatic variants, nucleotide transition C > A, and nucleotide transition C > G were classified as high or low groups. Patients were then classified into four groups based on their molecular characteristics: Group 1 included patients with high numbers of unfiltered somatic variants (greater than 570 variants per sample), high nucleotide transition C > A (greater than 9 substitutions per sample), and low nucleotide shift C > G (less than 1 substitution per sample). Group 2 included patients with microsatellite instability (any somatic mutation in a *MSH2*, *MSH6*, *MLH1*, and *PMS2*); group 3 included patients with *TP53* mutations and no mutation in *MSH2, MSH6, MLH1, PMS2*; all other patients were classified as group 4. All analyses were performed using SAS 9.4 (SAS Institute Inc., Cary, North Carolina, USA).

### NGS data analysis: identification of pathogenic and candidate somatic variants

In order to be inclusive of all potentially pathogenic variants, we performed a single sample workflow analysis of each T or NT sample using the Ion Reporter Software, with standard company filtering parameters. The results of these analyses were manually curated to identify somatic variants unique to the tumor specimen. The unfiltered results from the single sample and two sample workflow analyses were combined into a single list of potential somatic variants. Variants that were called by only one of the Ion Reporter workflows were manually verified in the original Ion Torrent Variant Caller output to validate the quality of the read. Variants identified by only one Ion Reporter workflow and not reported by the Ion Torrent Variant Caller were not felt to warrant further investigation. Using the Broad Institute’s Integrative Genomics Viewer (IGV), remaining variants were visually inspected at the sequence level to assess the quality of variant call and variants of poor quality were excluded [17]. Variants from the corresponding NT specimen were similarly inspected in IGV and if visually present, the variant was deemed germline and excluded. Lastly, consistent filtering parameters were applied to identify somatic variants of potential pathogenic interest removing variants with *p*-value > 0.05, minor allele frequency > 1%, and synonymous amino acid exchange (synonymous variants examined separately, see below).

Filtered variants were annotated to assess their pathogenic impact. The pathogenicity of amino acid changes resulting from non-synonymous single nucleotide variants was evaluated using tools of population genetics including Sorting Intolerant from Tolerant (SIFT) and Polymorphism Phenotyping v2 (PolyPhen-2). SIFT scores < 0.05 imply deleterious function. PolyPhen-2 scores range 0–1; though no strict cut-off delineates deleterious from benign function higher scores imply deleterious function. Variants predicted to be benign (SIFT > 0.05 and PolyPhen-2 < 0.5) were filtered out. To ensure that information of potential biological value was retained, variants with discordant SIFT and PolyPhen pathogenicity prediction scores were retained in the database for further investigation. Remaining variants were classified as single nucleotide variants (filtered somatic SNVs*)* and INDELs (filtered somatic INDELs). ClinVar was queried to identify variants in our dataset that have previously been reported as pathogenic. For those variants with unknown pathogenic impact, we collated functional information (gene, variant type and function, gene region) and SIFT/PolyPhen-2 predicted pathogenicity. Lastly, we interrogated TCGA and Catalog of Somatic Mutations in Cancer (COSMIC) databases to evaluate the frequency at which these mutations had been reported in association with malignancy. This data was used to classify somatic variants as pathogenic or as candidate variants of potential pathogenic interest. Somatic variants were designated pathogenic if they had been previously reported pathogenic in ClinVar [18] or had been documented in association with greater than 500 cancers in COSMIC. Somatic variants were designated candidate variants of potential pathogenic interest if predicted pathogenic by tools of population genetics (SIFT, PolyPhen) or had been documented in association with fewer than 500 cancers in COSMIC. The remaining variants without annotative data are deemed variants of uncertain significance.

Insertions and deletions (INDELs) were examined for pathogenic impact using the DataBase of Cancer Driver InDels (dbCID) [19].

Synonymous single nucleotide variants were examined for pathogenic impact using PrDSM (Prediction of Deleterious Synonymous Mutations) v1.0 [20] which averages the pathogenic rating generated by the three most accurate predictors for synonymous mutations [Transcript inferred Pathogenicity Score (TraP Score), Silent Variant Analyzer (SilVA score), and Functional Analysis through Hidden Markov Models (FATHMM-MKL score)]. By convention, PrDSM scores > 0.308 are suggestive of deleterious function.

For the analysis of *POLE* sequencing results, the resultant Ion Proton-generated BAM files containing identified variants were uploaded to Ion Reporter v5.6 and analyzed using both a single sample germline and a single sample somatic workflow in Ion Reporter, under the default parameters for each workflow. As there were fewer overall variants identified by the *POLE* sequencing, fewer filtering parameters were employed prior to visual examination of the variants. All variants, from each workflow, with a *p*-value of ≤ 0.05 were visually examined using IGV version 5.2.1 and those passing visual examination that were identified in exonic regions and with non-synonymous variant effects were investigated further by interrogating ClinVar [18], TCGA [21] through cBioPortal [22], and COSMIC [23] for previously reported assertions of pathogenicity and other variant effects.

### Clinical data analysis

Clinical data was abstracted from medical records including patient age at diagnosis, parity, race and ethnicity, pregnancy history, and tobacco use. Pathologic data was obtained including stage of disease, percentage myometrial invasion, presence of lymphovascular space invasion, and site of metastatic disease (including pelvic lymph nodes, para-aortic lymph nodes, omentum, ovaries, fallopian tubes, cervix or positive cytology). It was noted whether the patient received adjuvant treatment including chemotherapy, brachytherapy, or external beam radiation. Progression free survival was calculated in months from time of diagnosis to recurrence or progression of disease.

Data analysis was performed using Stata version 14.2 (StataCorp. 2015. *Stata Statistical Software: Release 14*. College Station, TX: StataCorp LP). Normality of continuous variables was visually assessed and if no substantial violations were noted, data was reported as means ± standard deviations. Otherwise, they were reported as medians with interquartile ranges (IQR). Categorical data was presented as number of subjects with percentages. Bivariate analysis was performed to assess the association between clinical and pathologic variables and USC relative to EAC. Continuous variables were assessed using two sample t-test or the Mann–Whitney U test, whereas categorical and dichotomous variables were examined using the chi-squared and Fisher’s exact tests, as appropriate. Univariate logistic regression was performed to assess the association of individual variables with USC. Odds ratios were reported with 95% confidence intervals (CI).

## Results

### Clinicopathologic variables of patients’ cohort

Forty-seven T and matched NT tissue specimens were sequenced for the targeted analysis of 156 cancer genes using the Einstein Custom Cancer Panel (ECCP) and the Ion Ampliseq Technology [16]. Clinical data for the 47 sequenced endometrial cancer samples is presented in Table 1. Most of the patients included in the study were of non-white race, representative of the patient population served by Montefiore Medical Center. Six of the evaluated patients had a history of prior malignancy: 4 had breast cancer, 1 lung cancer, and 1 with both breast cancer and leukemia. Following a diagnosis of endometrial cancer, all patients were treated with total hysterectomy and bilateral salpingo-oophorectomy. All but two patients in the grade 1 EAC cohort and one patient in the USC cohort underwent pelvic lymphadenectomy. Para-aortic lymph node sampling was performed in more than 50% of the patients (58% grade 1 EAC, 50% grade 2 EAC, 58% grade 3 EAC, and 85% USC). Patients in the USC cohort were more likely to have advanced (stage III-IV) disease than those in the EAC. More patients with USC than EAC received adjuvant chemotherapy, external beam radiation therapy, and brachytherapy. Progression free survival was significantly worse amongst patients with USC than those with EAC.

**Table 1:** Clinical and pathologic data stratified by endometrioid versus serous histology

	All (N = 47)	EAC (N = 34)	USC (N = 13)	OR (95% CI)^b^	*p*-value
Age (years)	63.6 ± 11.0	60.9 ± 11.2	70.7 ± 7.0		< 0.01
Parity (number of children)	2 (1, 4)	1 (0,3)	4 (2, 7)		< 0.01
Race/ethnicity^a^					
White	13 (27.7)	11 (32.4)	2 (15.4)	REF	0.08
Black	14 (29.8)	6 (17.7)	8 (61.5)	7.33 (1.16–46.23)	
Asian	3 (6.4)	3 (8.8)	0 (0)	N/A	
Hispanic	8 (19.2)	7 (20.6)	2 (15.4)	1.57 (0.18–13.86)	
Race/ethnicity^a^					
White	13 (33.3)	11 (40.7)	2 (16.7)	REF	0.13
Non-White	26 (66.7)	16 (59.3)	10 (83.3)	3.44 (0.63–18.84)	
Pregnancy history					
Nulliparous	11 (23.4)	11 (32.4)	0 (0)	REF	0.02
Parous	36 (76.7)	23 (63.9)	13 (100.0)	N/A	
Personal history of malignancy	6 (12.8)	3 (8.82)	3 (23.1)	3.10 (0.54–18.87)	0.33
Family history of malignancy	29 (61.7)	22 (64.7)	7 (53.9)	0.64 (0.17–2.32)	0.52
Tobacco use					
Never	35 (74.5)	24 (70.6)	11 (84.6)	REF	0.62
Former	9 (19.2)	7 (20.6)	2 (15.4)	0.62 (0.11–3.50)	
Current	3 (6.4)	3 (8.8)	0 (0)	N/A	
Metastatic disease					
Pelvic lymph nodes	9 (19.2)	4 (11.8)	5 (38.5)	5.00 (1.06–23.65)	0.09
Para-aortic lymph nodes	8 (17.0)	2 (5.9)	6 (46.2)	8.50 (1.33–54.12)	< 0.01
Omentum	0 (0)	0 (0)	0 (0)	N/A	N/A
Ovaries	6 (12.8)	4 (11.8)	2 (15.4)	1.50 (0.24–9.47)	0.74
Fallopian tubes	2 (4.3)	0 (0)	2 (15.4)	N/A	0.07
Cervix	17 (36.2)	7 (20.6)	10 (76.9)	12.86 (2.77–59.66)	< 0.01
Lower uterine segment	2 (4.3)	2 (5.8)	0 (0)	N/A	> 0.90
Lymphovascular invasion	18 (38.3)	11 (32.4)	7 (53.9)	2.44 (0.66–9.00)	0.18
Cytology	1 (2.1)	1 (2.9)	0 (0)	N/A	> 0.90
Myometrial invasion					
0%	5 (10.6)	5 (14.7)	0 (0)	REF	0.25
1–33%	18 (38.3)	13 (38.2)	5 (38.5)	0.38 (0.08–1.93)	
34–66%	14 (29.8)	11 (32.4)	3 (23.1)	0.27 (0.05–1.62)	
67–100%	10 (21.3)	5 (14.7)	5 (38.5)	N/A	
Stage					
I	23 (48.9)	22 (64.7)	1 (7.7)	REF	< 0.01
II	8 (17.0)	4 (11.8)	4 (30.8)	22.00 (1.92–251.54)	
III	15 (31.9)	7 (20.6)	8 (61.5)	25.14 (2.66–237.62)	
IV	1 (2.1)	1 (2.9)	0 (0)	N/A	
Stage					
I–II	31 (66.0)	26 (76.5)	5 (38.5)	REF	0.02
III–IV	16 (34.0)	8 (23.5)	8 (61.5)	5.20 (1.32–20.46)	
Treatment					
Chemotherapy	25 (53.2)	12 (35.3)	13 (100.0)	N/A	< 0.01
External beam radiation therapy	22 (46.8)	11 (32.4)	11 (84.6)	11.50 (2.17–61.04)	< 0.01
Brachytherapy	24 (51.1)	14 (41.2)	10 (76.9)	4.76 (1.11–20.50)	0.03
Recurrence or progression	15 (31.9)	7 (20.6)	8 (61.5)	6.17 (1.53–24.84)	0.01
Progression free survival (months)	33 (15, 45)	37.5 (24, 48)	15 (5, 26)		< 0.01
Vital status					
Alive	34 (72.3)	27 (79.4)	7 (53.9)	REF	0.06
Dead	4 (8.5)	1 (2.94)	3 (23.1)	11.57 (1.04–128.97)	
Lost to follow-up	9 (19.2)	6 (17.7)	3 (23.1)	1.93 (0.38–9.71)	

Data with plus-minus values represent means ± standard deviation, otherwise reported as median (interquartile range). Categorical data are presented as N (%)

*EBRT* external beam radiation therapy, *LVSI *positive lymphovascular space invasion

^a^Based on 39 patients with available race/ethnicity information

^b^Refers to the odds of individual covariates being associated with serous histology relative to endometrioid histology. N/A values indicate that odds ratios are incalculable as subgroups predict failure perfectly

### Endometrioid and serous histology present distinctive molecular features

Endometrioid tumors had a significantly higher number of pathogenic somatic variants plus candidate somatic variants of potential pathogenic interest than serous cancers (Table 2). The frequency of *PTEN* and *PIK3CA* SNVs was significantly higher for EAC compared with USC and the frequency of *TP53* SNVs was significantly higher in USC than EAC. Further division of EAC by grade (1, 2, or 3) demonstrated differences in the number of pathogenic somatic variants and candidate somatic variants of potential pathogenic interest (Table 3). The grade 3 EAC cohort had significantly more pathogenic somatic variants plus candidate somatic variants of potential pathogenic interest compared with USC, grade 1 EAC, and grade 2 EAC. Raw data by sample is presented in Additional file 2: Table S2. No significant racial differences were identified in the number of unfiltered somatic variants, unfiltered somatic SNVs, pathogenic somatic variants, candidate somatic variants of potential pathogenic interest, or pathogenic somatic variants plus candidate somatic variants of potential pathogenic interest (Additional file 3: Table S3). A single nucleotide variant at *PTEN* locus chr10:89692904 was significantly (*p* = 0.02) associated with grade 1 EAC (42%) compared with grade 2 EAC (10%), grade 3 EAC (8%), and USC (0%) (Additional file 4: Table S4). A summary of filtered somatic variants including pathogenic variants, candidate variants of potential pathogenic interest, and variants of uncertain significance with annotative data for each sample is included in Additional file 5: Table S5.

**Table 2:** Number of variants stratified by histologic subtype

	All (N = 47)	EAC (N = 34)	USC (N = 13)	*p*-value
Unfiltered somatic variants	67 (57, 86)	71 (60, 90)	63 (56, 72)	0.11
Unfiltered somatic SNVs	63 (54, 79)	65 (55, 85)	58 (53, 69)	0.19
SNV C > A	6 (4, 9)	6.5 (4, 9)	5 (4, 8)	0.41
SNV C > G	2 (1, 3)	2 (1, 3)	2 (2, 3)	0.33
SNV C > T	9 (5, 13)	10 (8, 15)	6 (5, 7)	< 0.01
SNV T > A	4 (2, 6)	4 (2, 6)	4 (3, 5)	0.75
SNV T > C	7 (4, 10)	7 (4, 11)	5 (4, 8)	0.35
SNV T > G	4 (3, 7)	4 (3, 7)	4 (3, 6)	0.39
Filtered somatic SNVs	8 (4, 14)	9 (7, 16)	3 (3, 8)	< 0.01
Filtered somatic INDELs	2 (0, 3)	2 (1, 3)	1 (0, 3)	0.33
Pathogenic somatic variants	1 (0, 2)	1 (1,2)	0 (0, 1)	0.06
Candidate somatic variants of potential pathogenic interest	5 (3, 10)	6 (4, 13)	2 (2, 3)	< 0.01
Pathogenic somatic variants *plus* candidate somatic variants of potential pathogenic interest	6 (3, 10)	7 (5, 14)	3 (2, 4)	< 0.01
PTEN mutation [N(%)]	30 (63.8)	28 (82.4)	2 (15.4)	< 0.01
PIK3CA mutation [N(%)]	28 (59.6)	25 (73.5)	3 (23.1)	< 0.01
TP53 mutation [N(%)]	16 (34.0)	6 (17.7)	10 (76.9)	< 0.01

**Table 3:** Number of variants stratified by histologic subtype and grade

	All (N = 47)	Grade 1 EAC (N = 12)	Grade 2 EAC (N = 10)	Grade 3 EAC (N = 12)	USC (N = 13)	*p*-value
Unfiltered somatic variants	67 (57, 86)	64.5 (55.5, 71.5)	74.5 (61, 82)	139 (66, 346.5)	63 (56, 72)	0.03
Unfiltered somatic SNVs	63 (54, 79)	60 (52.5, 67.5)	72 (59, 79)	132 (58, 340)	58 (53, 69)	0.18
SNV C > A	6 (4, 9)	5.5 (3.5, 7.5)	5.5 (4, 8)	10.5 (5, 63)	5 (4, 8)	0.16
SNV C > G	2 (1, 3)	2.5 (1, 3.5)	1 (1, 2)	2.5 (1, 3)	2 (2, 3)	0.39
SNV C > T	9 (5, 13)	9.5 (8.5, 12.5)	8.5 (5, 12)	15.5 (9, 49.5)	6 (5, 7)	< 0.01
SNV T > A	4 (2, 6)	3 (2.5, 4)	5.5 (2, 7)	4.5 (2.5, 6.5)	4 (3, 5)	0.33
SNV T > C	7 (4, 10)	5.5 (3, 6.5)	7.5 (6, 12)	10.5 (7, 19.5)	5 (4, 8)	0.04
SNV T > G	4 (3, 7)	4 (2, 4)	7 (5, 8)	5.5 (3, 18)	4 (3, 6)	0.08
Filtered somatic SNVs	8 (4, 14)	8 (7, 9)	7 (5, 16)	56.5 (8.5, 202.5)	3 (3, 8)	< 0.01
Filtered somatic INDELs	2 (0, 3)	1 (1, 2)	1.5 (1, 3)	2.5 (1.5, 4)	1 (0, 3)	0.36
Pathogenic somatic variants	1 (0, 2)	1 (1, 2)	0.5 (0, 1)	1.5 (1, 3)	0 (0, 1)	< 0.01
Candidate somatic variants of potential pathogenic interest	5 (3, 10)	5 (4, 7)	4 (4, 13)	25 (6, 89.5)	2 (2, 3)	< 0.01
Pathogenic somatic variants *plus* candidate somatic variants of potential pathogenic interest	6 (3, 10)	6.5 (5, 7.5)	5 (4, 14)	26.5 (7, 92.5)	3 (2, 4)	< 0.01
PTEN mutation [N(%)]	30 (63.8)	10 (83.3)	7 (70.0)	11 (91.7)	2 (15.4)	< 0.01
PIK3CA mutation [N(%)]	28 (59.6)	8 (66.7)	7 (70.0)	10 (83.3)	3 (23.1)	0.01
TP53 mutation [N(%)]	16 (34.0)	0 (0)	2 (20)	4 (33.3)	10 (76.9)	< 0.01

### Examination of synonymous single nucleotide variants

520 unique synonymous variants were found in 47 endometrial cancer specimens. Of these, 106 are predicted deleterious by PrDSM with pathogenicity scores ranging 0.308 to 0.830. The clinical utility and relevance of synonymous variants warrants further investigation. These synonymous variants with associated TraP, SilVA, FATHMM-MLK, and PrDSM scores are included in Additional file 6: Table S6.

### Examination of INDELs

203 unique insertion/deletion variants were identified (Additional file 7: Table S7). Of these, 2 INDELs are identified in dbCID in association with cancer (*PTEN* frameshift c.950_953delTACT previously reported with endometrial cancer and *PIK3CA* non-frameshift c.325_327delGAA previously associated with melanoma). Five additional INDELs in *PIK3R1, TP53,* and *PTEN* are reported in overlapping but non-identical loci in dbCID in association with cancer.

### Targeted sequencing identifies hypermutated samples based on somatic mutation load

Simple visual inspection of the number of unfiltered somatic SNVs (Additional file 2: Table S2) in each sample revealed a subset of six grade 3 EACs with high tumor mutational burden (184–581 unfiltered somatic SNVs, median 340 IQR 193,399) (Table 4). The remaining 41 samples had significantly fewer number of unfiltered somatic SNVs ranging 29–311 (median 61 IQR 53.73; *p* < 0.01), except for two samples with 249 and 311 unfiltered somatic SNVs. However, these samples were excluded from the hypermutated group because when inspected visually in IGV, most of the variants were found to be the result of sequencing error.

**Table 4:** Number of variants stratified by low versus high tumor mutational burden

	All (N = 47)	Non-hypermutated (N = 41)	Hypermutated (N = 6)	*p*-value
Unfiltered somatic variants	67 (57, 86)	65 (56, 76)	346.5 (209, 402)	< 0.01
Unfiltered somatic SNVs	63 (54, 79)	61 (53, 73)	340 (193, 399)	< 0.01
SNV C > A	6 (4, 9)	5 (4, 8)	63 (37, 81)	< 0.01
SNV C > G	2 (1, 3)	2 (1, 3)	3 (2, 3)	0.58
SNV C > T	9 (5, 13)	8 (5, 12)	49.5 (31, 95)	< 0.01
SNV T > A	4 (2, 6)	4 (2, 5)	5.5 (4, 7)	0.30
SNV T > C	7 (4, 10)	6 (4, 8)	19.5 (15, 22)	< 0.01
SNV T > G	4 (3, 7)	4 (3, 6)	18 (11, 27)	< 0.01
Filtered somatic SNVs	8 (4, 14)	7 (4, 9)	202.5 (105, 234)	< 0.01
Filtered somatic INDELs	2 (0, 3)	2 (0, 3)	2 (1, 3)	0.57
Pathogenic somatic variants	1 (0, 2)	1 (0, 1)	3 (2, 6)	< 0.01
Candidate somatic variants of potential pathogenic interest	5 (3, 10)	4 (2, 7)	89.5 (43, 119)	< 0.01
Pathogenic somatic variants *plus* candidate somatic variants of potential pathogenic interest	6 (3, 10)	5 (3, 8)	92.5 (45, 125)	< 0.01

To further explore the hypermutated samples, the 6 hypermutated endometrial tumor samples, 4 endometrial tumor samples not hypermutated including one with unfiltered somatic SNVs overlapping the range for the hypermutated group, and 2 non-cancer controls (total of 12 samples) underwent *POLE* sequencing (Table 5). Of these, one of the non-cancer controls and two non-hypermutated endometrial cancer samples each had one exonic variant (p.Asn1396Ser or p.Leu2274Val, rs5744934 or rs148788180, respectively) mapping to the *POLE* catalytic domain but both are reported benign in ClinVar [24]. Both variants are predicted benign by PolypPhen. Interestingly, pAsn1396Ser is predicted pathogenic by SIFT. Of the six hypermutated samples, four exhibited a missense mutation p.Pro286Arg, which is reported as a recurrent mutation in the provisional endometrial dataset in TCGA (20 out of 509 cases or 4%) and found in colorectal cancer as well [25]. In one sample, we mapped the p.Val411Leu variant, also classified as pathogenic and recurrent in the provisional endometrial dataset in TCGA (13 out of 509 cases or 2.5%). In the remaining hypermutated sample, we mapped a stoploss mutation outside the known functional domains mapping at the 3′ end of the gene (p.Ter2287Trp). However, this mutation has not been reported in association with disease. Additionally, all of the hypermutated samples contained other exonic *POLE* variants of both benign and uncertain impact, occurring both within and outside functional domains of the *POLE* gene, whereas the non-cancer control and the non-hypermutated endometrial cancer samples each had only a single benign mutation.

**Table 5:** Mutations mapping to POLE

Amino acid change	Coding	EC hypermutated						EC NON hypermutated				Control blood	
p.Ter2287Trp	c.6860A > G			✓									
p.Leu2274Val	c.6820C > G										✓		
p.Glu2140Lys	c.6418G > A						✓						
p.Ile1864Ser	c.5591 T > G				✓								
p.Asp1211Gly	c.3632A > G						✓						
p.Asp530Asn	c.1588G > A						✓						
p.Ala456Val	c.1367C > T				✓								
p.Val411Leu	c.1231G > T						✓						
p.Asn151Lys	c.453 T > G				✓								
p.Leu98Val	c.292 T > G	✓											
p.Ala252Val	c.755C > T		✓	✓									
p.Asn1396Ser	c.4187A > G		✓							✓		✓	
p.Pro286Arg	c.857C > G	✓	✓		✓✓	✓							
Pathogenic variants		5						0				0	
Unknown significance in functional domains		3						0				0	
Unknown significance outside functional domains		4						0				0	
Benign in functional domains		4						2				0	
TOT number exonic variants		16						2				1	

Of the six hypermutated samples, five had mutations in one or more mismatch repair genes including *MLH1, PMS2, MSH2,* and *MSH6* (Additional file 2: Table S2). In three of these samples, the mutations were characterized as candidate variants of potential pathogenic interest based on functional prediction scores. Two samples carried a p.Glu580Ter variant in *MSH2* which is predicted pathogenic by ClinVar. This variant was reported in 1 of 509 samples in the provisional endometrial dataset in TCGA as well as four cancers (one endometrial) in COSMIC.

Examining the clinical characteristics of these six hypermutated samples with *POLE* mutations, all were found to be grade 3 EAC. These six hypermutated grade 3 EACs had significantly more unfiltered somatic SNVs (median 340 IQR 193,399) than the remaining six grade 3 EACs (median 58 IQR 40.63) analyzed in our cohort (*p* < 0.01). Genetically distinct, these two groups were compared clinically. Three patients in each group presented with stage I disease. However, patients with stage I disease in the low tumor mutational burden group were more likely to receive adjuvant treatment (chemotherapy and/or radiation) based on uterine prognostic factors (lymphovascular invasion, myometrial invasion). Based on these uterine factors and stage, all patients in the non-hypermutated group were recommended to receive adjuvant chemotherapy and radiation (1 declined). In comparison, three patients in the hypermutated group received adjuvant treatment (1 chemotherapy alone, 1 chemotherapy and radiation, 1 radiation alone) based on stage. For patients with stage I disease in the hypermutated group, none received adjuvant treatment based on the uterine factors. All but one patient with hypermutated grade 3 EAC were alive without evidence of recurrent disease. None of the patients with *POLE* mutations had a personal history of a secondary malignancy.

All 47 endometrial cancer samples were analyzed to determine if targeted next generation sequencing alone could produce a similar clustering hierarchy as TCGA (*POLE* ultra-mutated, MSI hypermutated, copy number low, and copy number high). We identified six samples with high tumor mutational burden and *POLE* mutation consistent with TCGA group 1. We do not have MMR protein expression data for these patients as MMR immunohistochemistry (IHC) was not part of routine endometrial cancer evaluation at the time that these specimens were collected. Excluding those with *POLE* mutations or high mutational burden, none of the remaining samples were found to have pathogenic somatic variants or candidate variants of potential pathogenic interest in one of four DNA MMR proteins (*MLH1, MSH2, MSH6,* and *PMS2*). Of the remaining 41 samples, we evaluated the presence of a mutation in the PI3-Kinase pathway (*PTEN, PIK3CA, PIK3R1, PIK3R3*) and *TP53* (Additional file 2: Table S2). Twenty-six samples had mutations in the PI3-Kinase pathway without associated *TP53* mutation. Of these, 25 (96%) were EAC. All six of the grade 3 EACs without *POLE* mutation and high tumor mutational burden demonstrated this genetic pattern. Nine samples had mutations in *TP53* without a mutation in the PI3-Kinase pathway genes. Of these, eight (89%) were USC. Unexpectedly, the remaining 12 samples demonstrated both *TP53* and *PTEN/PIK3* mutation (n = 7), or no *TP53* or *PTEN/PIK3* mutation (n = 5). Four of these samples were USC (33%) and grade 3 EAC (33%) and 2 were grade 1 (17%) or grade 2 (17%) EAC tumors. Conclusions regarding clinical phenotype of these 12 samples is unattainable due to low numbers.

## Discussion

In this study, we sought to determine whether a custom-designed targeted next generation sequencing panel could be utilized to classify 47 endometrial cancers into the four molecular groups defined by TCGA (*POLE* ultra-mutated, MSI hypermutated, copy number low, copy number high). When this in-house custom panel was designed, the value of *POLE* in molecular classification of endometrial cancer was not understood and *POLE* was not included in the panel design. However, targeted panel sequencing did identify six cases of high tumor mutational burden for which subsequent *POLE* sequencing confirmed the presence of *POLE* mutation. Next generation sequencing may be sufficient to replace whole exome sequencing, as performed by TCGA, as a means of measuring tumor mutational burden. Previously, authors have reported that 4–7% of endometrial cancers are included in the *POLE* molecular subgroup [13, 26]. We identified p.Pro286Arg and p.Val411Leu hotspot mutations in *POLE* in five (83%) of these cases. This is consistent with two prior studies that identified these two hotspot variants in 65–76% *POLE* ultra-mutated endometrial cancers [13, 26]. Moreover, both variants are also present in the COSMIC database with pathogenic Functional Analysis Through Hidden Markov Models (FATHMM) predictions. Endometrium is highly represented in the distribution of tissues in which these variants occur. Eight-two percent of the entries for the p.Pro286Val variant occurred in endometrial tissue and 73% of p.Val411Leu entries originate from endometrium. Both variants map to the exonuclease domain of the gene. In the remaining sample with *POLE* mutation and high tumor mutational burden, we identified a stoploss mutation at position 2287, near the 3′ end of the gene and outside of the known functional domains. In TCGA, only one stoploss mutation was identified in the *POLE* ultra-mutated molecular subgroup at position 1930, in a domain of unknown function. Interestingly, targeted next generation sequencing of this sample also identified a p.Asp316Asn variant in the *POLD1* gene. This gene codes for the DNA polymerase delta 1 catalytic subunit. While reported as a variant of uncertain significance in ClinVar, this variant does occur in the exonuclease 1 domain of *POLD1* and has been predicted to be pathogenic by the in silico tools SIFT and PolyPhen. Germline exonuclease domain mutations have been shown to predispose to both endometrial and colorectal cancers [27]. This specific amino acid change has also been previously associated with hypermutation in cancer [28]. Furthermore, other protein changes at this position are classified as pathogenic by Bellido et al. [29].

There is a high rate of concomitant *POLE* mutation and mutation in MMR gene. Of the six *POLE* ultra-mutated samples, five (83%) had pathogenic somatic variants or candidate somatic variants of potential pathogenic interest in a mismatch repair gene. One sample had candidate variants in *MLH1, PMS2, MSH2*, and *MSH6*; two had a pathogenic variant in *MSH2*; and the remaining 2 had candidate variants in *MLH1* and *MSH6*, respectively. In TCGA dataset, 63% of samples with a *POLE* mutation also demonstrated a mutation in a MMR gene [13]. Similarly, Cosgrove et al. [26] reported *POLE* mutations in 5% of MMR deficient samples . Given the overlap of *POLE* and MMR mutations in published data and our reported cases, we acknowledge the possibility that our six samples with high tumor mutational burden may classify into either *POLE* ultra-mutated or MSI hypermutated subgroups with additional supporting data not measured by next generation sequencing. A previously published study evaluating comprehensive genomic profiling through Foundation Medicine identified high tumor mutational burden in association with one *POLE* tumor, 8 MSI hypermutated tumors, and one copy number low tumor [30]. Given the frequency of p.Pro286Arg and p.Val411Leu in our subset of cases with high tumor mutational burden and the association between these hotspot mutations and *POLE* ultra-mutated tumors, it is more likely that these tumors represent a *POLE* ultra-mutated subgroup.

We identified no patients in our cohort with MMR mutations in the absence of *POLE* mutations. Previously published studies report approximately 40% of endometrial cancers subgroup into the MSI hypermutated molecular class. These differences are likely the result of the method of microsatellite instability measurement. In TCGA study, microsatellite instability was measured using PCR amplification at seven repeat loci [13]. In an analysis of GOG 210, Cosgrove et al. [26] measured MMR defects using a combination of microsatellite instability, MMR immunohistochemistry, and *MLH1* hypermethylation. This highlights the understanding that somatic variants in mismatch repair genes are one of multiple mechanisms resulting in microsatellite instability. Epigenetic alterations, such as hypermethylation, and frameshift mutations resulting in deficient MMR protein expression as measured by immunohistochemistry contribute significantly to the MSI hypermutated molecular group. In TCGA, most tumors in the MSI hypermutated group demonstrated *MLH1* promoter hypermethylation. A 1997 National Cancer Institute (NCI) workshop on microsatellite instability for cancer detection validated and recommended a panel of five microsatellites as a reference panel for research in the field [31]. From this, we conclude that true MSI testing, as recommended by the NCI, is required to accurately identify an MSI hypermutated group and that next generation sequencing with identification of single nucleotide variants is insufficient.

TCGA used the presence of copy number alterations to define the last two molecular clusters of endometrial cancer—copy number low and copy number high. The copy number high group consisted of 94% of USC and 12% of EAC, including 24% of grade 3 EAC [13]. Ninety percent of the patients in this cohort had *TP53* mutations leading subsequent authors to investigate the utility of *TP53* as a surrogate for copy number alterations. After identification of MSI (MMR IHC) and *POLE* (NGS) groups, Talhouk et all used *p53* IHC as a surrogate for copy number alterations to distinguish the remaining two molecular cohorts (*TP53* wild type and *TP53* abnormal expression). Classification of endometrial cancers according to this methodology produced survival curves similar to those described by TCGA [32]. They replicated these findings in a larger confirmatory study [33]. Cosgrove et al. evaluated the *TP53* status (both NGS and protein expression by IHC) in 20 samples from each molecular subgroup and reported that only 55% of the copy number altered tumors had a *TP53* abnormality and that 5% of the copy number stable groups harbored a *TP53* abnormality [26]. Based on the ambiguity of these studies, we did not feel confident in the adequacy of *TP53* to serve as a surrogate for the copy number status in our study.

Lastly, while the majority of samples in our study classified genomically as *POLE* ultra-mutated (n = 6, 100% grade 3 EAC), *TP53* mutated (n = 9, 89% USC), or PI3-Kinase pathway mutated (n = 26, 96% EAC), we did identify a subset of seven cases with both *TP53* and *PTEN/PIK3* mutation (1 grade 2 EAC, 4 grade 3 EAC, 2 USC). Like the authors of TCGA landmark publication, we conclude that there is a subset of endometrial tumors with unique genetic patterns that do not align with the traditionally described relationships between histology and genetic profiling.

TCGA investigation published a landmark paper in 2013 describing four molecular classes of endometrial cancer (*POLE* ultra-mutated, MSI hypermutated, copy number high, copy number low) based on comprehensive and integrated molecular profiling involving six genomic and proteomic platforms. These four clusters are associated with survival differences, superior survival seen in the *POLE* ultra-mutated group and inferior survival in the copy number high group. Since that time, authors have sought to identify algorithms to define these four groups using more clinically accessible methods of investigation. Cosgrove et al. analyzed 1040 specimens collected as part of GOG 210 protocol. MMR defects (as identified through microsatellite instability testing, mismatch repair protein expression/IHC, and *MLH1* hypermethylation) were defined first. For microsatellite stable cases, copy number status was then assigned using loss of heterozygosity at 3 microsatellite repeats. Finally, *POLE* mutational status was assessed for copy number low cases. Ninety-five percent of cases were successfully classified into 1 of 4 groups based on this algorithm. The resultant survival curves for these groups mirrored those seen in TCGA, with the highest risk of cancer-specific death (19%) in the copy number high group and lowest risk of cancer-specific death (2.6%) in the *POLE* group [26]. Talhouk et al. utilized a similar, albeit different, algorithm to produce similar survival curves. They identified MSI hypermutated and *POLE* groups utilizing mismatch repair protein expression (immunohistochemistry) and *POLE* sequencing followed by *TP53* IHC to distinguish *TP53* wild type (copy number low) and *TP53* abnormal (copy number high) groups. In each of these studies, clinically available testing was applied to categorize cases into one of four molecular classes with high efficiency. However, each of these studies required multi-platform testing—microsatellite instability/loss of heterozygosity, immunohistochemistry, hypermethylation in addition to next generation sequencing. In our study of next generation sequencing alone, we could not confidently reproduce these four groups. Our data suggest that targeted next generation sequencing alone may identify *POLE* ultramutated tumors on the basis of high mutational burden but is otherwise inadequate to assign a molecular classification to endometrial cancer.

## Conclusions

TCGA investigation published a landmark paper in 2013 describing four molecular classes of endometrial cancer (*POLE* ultra-mutated, MSI hypermutated, copy number high, copy number low) based on comprehensive and integrated molecular profiling involving six genomic and proteomic platforms. Although WGS and WES, as utilized by TCGA, provide power to dissect the complexity of the whole cancer genome, the application of these approaches in the clinical setting is limited by sequencing cost and time. Subsequent publications have described algorithms to define these four groups using more clinically accessible methods of investigation with high efficiency. However, these studies utilized multi-platform testing—microsatellite instability/loss of heterozygosity, immunohistochemistry, hypermethylation in addition to next generation sequencing. Unfortunately, in our study of next generation sequencing alone, we could not confidently reproduce these four groups. We did identify that high mutational burden predicts the presence of *POLE* mutation with high accuracy. Otherwise, our data suggest that targeted next generation sequencing is inadequate to assign a molecular classification to endometrial cancer. In the current era of targeted cancer care, further investigation is required to improve the efficiency and accessibility of genomic profiling such that genomic data can be utilized to individualize adjuvant treatment decisions.

## Supplementary information

**Additional file 1.** **Table S1** - Panel design for *POLE* sequencing.

**Additional file 2.** **Table S2** - Next generation sequencing data presented by sample.

**Additional file 3.** **Table S3** - Number of variants stratified by white vs non-white race.

**Additional file 4.** **Table S4** - Somatic variants compared by tumor histology and grade.

**Additional file 5.** **Table S5** - Filtered somatic variants identified in each sample with annotative data.

**Additional file 6.** **Table S6** - PrDSM pathogenicity scores for synonymous variants.

**Additional file 7.** **Table S7** - Insertion/deletion variants identified in each sample.

## Data Availability

The datasets used and/or analyzed during the current study are available from the corresponding author on reasonable request (genetic data output for each tumor and matched non-tumor sample). Summarized data of somatic variants from paired tumor and non-tumor samples are included in this published article and its supplementary information files. The below listed datasets were used for data analysis (weblinks provided). TCGA, COSMIC, ClinVar, IGV, dbCID, and PrDSM datasets were manually curated. Additional file 7: Table S7 includes data collated from SIFT, PolyPhen2, dbSNP, Grantham Score, 5000 Exomes Project, DGV, DRUGBANK, Gene Ontology, OMIM, Pfam, phyloP Score by the IonReporter pipeline. Additional file 6: Table S6 includes data collated from TraP, SilVA, and FATHMM-MKL by the PrDSM pipeline. TCGA: The Cancer Genome Atlas via cBioPortal for Cancer Genomics http://www.cbioportal.org hg19: hg19-Genome Reference Consortium GRCh37 (NCBI human reference genome) https://genome.ucsc.edu/cgi-bin/hgTracks?db=hg19&lastVirtModeType=default&lastVirtModeExtraState=&virtModeType=default&virtMode=0&nonVirtPosition=&position=chrX%3A15578261%2D15621068&hgsid=933363611_wbMezEdYiVDkycAcFS740NDN4lFQ IGV: Broad Institute’s Integrative Genomics Viewer http://software.broadinstitute.org/software/igv/ ClinVar https://www.ncbi.nlm.nih.gov/clinvar/ COSMIC: Catalog of Somatic Mutations in Cancer https://cancer.sanger.ac.uk/cosmic dbCID: DataBase of Cancer Driver InDels http://bioinfo.ahu.edu.cn:8080/dbCID/ PrDSM v1.0: Prediction of Deleterious Synonymous Mutations http://bioinfo.ahu.edu.cn:8080/PrDSM/ SIFT: Sorting Intolerant from Tolerant https://sift.bii.a-star.edu.sg/sift4g/ PolyPhen2: Prediction of functional effects of human nsSNPs http://genetics.bwh.harvard.edu/pph2/ dbSNP: The Short Genetic Variations (SNV) database http://www.ncbi.nlm.nih.gov/SNP/index.html Grantham Score Grantham R. Amino Acid Difference Formula to Help Explain Protein Evolution. *Science* 1974, 185(4154):862–864. 5000 Exomes Project Tennessen JA, Bigham AW, O'Connor TD, Fu W, Kenny EE, Gravel S, McGee S, Do R, Liu X, Jun G. et al. Evolution and functional impact of rare coding variation from deep sequencing of human exomes. *Science* 2012;5(6090):64–69 DGV: Database of Genomic Variants http://dgv.tcag.ca/dgv/app/home DRUGBANK https://go.drugbank.com GO: Gene Ontology Browser http://www.informatics.jax.org/vocab/gene_ontology/GO:0005730 OMIM: Online Mendelian Inheritance in Man https://www.ncbi.nlm.nih.gov/omim Pfam https://pfam.xfam.org phyloP Score: Phylogenetic Analysis with Space/Time Models http://compgen.cshl.edu/phast/resources.php Hubisz MJ, Pollard KS, Siepel A. PHAST and RPHAST: phylogenetic analysis with space/time models. *Brief Bioinform* 2011, 12(1):41–51. TraP: Transcript inferred Pathogenicity Score http://trap-score.org/about.jsp SilVA: SILVA rRNA Database Project https://www.arb-silva.de FATHMM-MKL: Predict the Functional Consequences of Non-Coding and Coding Single Nucleotide Variants (SNVs) http://fathmm.biocompute.org.uk/fathmmMKL.htm.

## Abbreviations

TCGA: The Cancer Genome Atlas
IQR: Interquartile range
EAC: Endometrioid endometrial cancer
USC: Uterine serous carcinoma
INDELs: Insertions and deletions
NGS: Next generation sequencing
WGS: Whole genome sequencing
WES: Whole exome sequencing
MMR: Mismatch repair
SNV: Single nucleotide variant
T: Tumor
NT: Non-tumor
H&E: Hematoxylin and eosin
ECCP: Einstein custom cancer panel
ISP: Ion sphere particles
Hg19: Human genome version 19
FFPE: Formalin-fixed paraffin embedded
TVC: Torrent variant caller
IGV: Integrated genomics viewer
SIFT: Sorting intolerant from tolerant
PolyPhen-2: Polymorphism phenotyping v2
COSMIC: Catalog of somatic mutations in cancer
CI: Confidence interval
MSI: Microsatellite instability
IHC: Immunohistochemistry
NCI: National Cancer Institute
