# Supplementary material for: Utility of a custom designed next generation DNA sequencing gene panel to molecularly classify endometrial cancers according to The Cancer Genome Atlas subgroups
Source: BMC Med Genomics. 2020 Nov 30;13:179. doi: 10.1186/s12920-020-00824-8 (PMC7706212; doi:10.1186/s12920-020-00824-8)
Supplement: Supplementary file 3 — Additional file 3. Table S3 - Number of variants stratified by white vs non-white race. [file 12920_2020_824_MOESM3_ESM.docx]

**Supplementary Table 3: Number of variants stratified by white vs. non-white race***

|  | All  (N=39) | White  (N= 13) | Non-White  (N = 26) | P-Value |
| --- | --- | --- | --- | --- |
| Unfiltered Somatic Variants | 66 (56, 86) | 65 (56, 89) | 66.5 (56, 82) | 0.79 |
| Unfiltered Somatic SNVs | 62 (50, 79) | 61 (49, 75) | 63.5 (53, 79) | 0.77 |
| SNV C>A | 6 (4, 9) | 7 (5, 9) | 5.5 (4, 9) | 0.35 |
| SNV C>G | 2 (1,3) | 3 (2,3) | 2 (1,3) | 0.07 |
| SNV C>T | 9 (5, 13) | 10 (8, 15) | 8 (5, 12) | 0.27 |
| SNV T>A | 4 (2, 6) | 4 (3, 6) | 3.5 (2, 6) | 0.79 |
| SNV T>C | 6 (4, 9) | 6 (4,8) | 6.5 (4,10) | 0.58 |
| SNV T>G | 4 (3, 7) | 4 (2, 6) | 4.5 (3, 7) | 0.67 |
| Filtered Somatic SNVs | 8 (4, 14) | 8 (7, 14) | 7.5 (3, 9) | 0.20 |
| Filtered Somatic INDELs | 2 (1, 3) | 1 (1, 3) | 2 (0, 3) | 0.67 |
| Pathogenic Somatic Variants | 1 (0, 1) | 1 (0, 2) | 1 (0, 1) | 0.65 |
| Candidate Somatic Variants of Potential Pathogenic Interest | 4 (2, 7) | 5 (3, 7) | 4 (2, 7) | 0.62 |
| Pathogenic Somatic Variants *plus* Candidate Somatic Variants of Potential Pathogenic Interest | 5 (2, 8) | 6 (3, 8) | 5 (2, 8) | 0.63 |

***Based on 39 patients who had complete racial/ ethnicity information evaluable**
